# Prognostic Role of Preoperative Sarcopenia Evaluation of Cervical Muscles with Long-Term Outcomes of Patients with Oral Squamous Cell Carcinoma

**DOI:** 10.3390/cancers13184725

**Published:** 2021-09-21

**Authors:** Takuya Yoshimura, Hajime Suzuki, Hirotaka Takayama, Shotaro Higashi, Yuka Hirano, Masahiro Tezuka, Takayuki Ishida, Kiyohide Ishihata, Marie Amitani, Haruka Amitani, Yasuhiro Nishi, Yasunori Nakamura, Yasushi Imamura, Etsuro Nozoe, Norifumi Nakamura

**Affiliations:** 1 Department of Oral and Maxillofacial Surgery, Kagoshima University Graduate School of Medical and Dental Sciences, Kagoshima 890-8520, Japan; y-taku@dent.kagoshima-u.ac.jp (T.Y.); h-tkym@d1.dent.kagoshima-u.ac.jp (H.T.); higashi@d1.dent.kagoshima-u.ac.jp (S.H.); y-hrn@d1.dent.kagoshima-u.ac.jp (Y.H.); tetsu@dent.kagoshima-u.ac.jp (M.T.); taka-isi@dent.kagoshima-u.ac.jp (T.I.); ishihata@dent.kagoshima-u.ac.jp (K.I.); nozoe@dent.kagoshima-u.ac.jp (E.N.); nakamura@dent.kagoshima-u.ac.jp (N.N.); 2 Department of Community-Based Medicine, Kagoshima University Graduate School of Medical and Dental Sciences, Kagoshima 890-8520, Japan; marisame@m3.kufm.kagoshima-u.ac.jp; 3 Department of Psychosomatic Internal Medicine, Kagoshima University Graduate School of Medical and Dental Sciences, Kagoshima 890-8520, Japan; amitani@m3.kufm.kagoshima-u.ac.jp; 4 Department of Oral and Maxillofacial Prosthodontics, Kagoshima University Graduate School of Medical and Dental Sciences, Kagoshima 890-8520, Japan; shar@dent.kagoshima-u.ac.jp; 5 Department of Oral Surgery, Kagoshima Medical Center, National Hospital Organization, Kagoshima 892-0853, Japan; nyasu@kagomc2.hosp.go.jp; 6 Department of Internal Medicine, Kagoshima Kouseiren Hospital, Kagoshima 890-0062, Japan; yasushii@po.synapse.ne.jp

**Keywords:** oral cancer, head and neck cancer, sarcopenia, computed tomography, cervical muscles, psoas muscle index, intramuscular adipose tissue content

## Abstract

**Simple Summary:**

As sarcopenia has been shown to be associated with a variety of functional impairments and increased mortality, it has become increasingly important to evaluate sarcopenia comorbidity in oral squamous cell carcinoma (OSCC). We assessed sarcopenia in patients with OSCC in a way that is feasible in daily practice and retrospectively evaluated its impact on prognosis. Patients with both low quality and low quantity of cervical muscles had a significantly worse prognosis. Assessing sarcopenia by evaluating both the quality and quantity of preoperative cervical skeletal muscle mass may be useful in optimizing the treatment of patients with OSCC without requiring additional imaging or patient burden.

**Abstract:**

Accumulating evidence has shown that sarcopenia in patients with oral squamous cell carcinoma (OSCC) is at a risk of poor prognosis. There is no universal consensus on how to assess sarcopenia in patients with OSCC in daily practice. It is important to validate the usefulness of sarcopenia assessment from cervical muscles, which are frequently used in routine clinical practice in patients with OSCC. In this study, we investigated whether preoperative lumbar (L3) skeletal muscle mass and adiposity in OSCC patients were associated with cervical (C3) skeletal muscle mass and adiposity from CT measurements. We also investigated whether skeletal muscle mass and adiposity in the C3 muscles were associated with survival rates in patients with OSCC. We demonstrated that both the quality and quantity of muscle between the C3 and L3 levels were positively correlated with each other. We also demonstrated that the survival rates in patients with low sternocleidomastoid muscle mass index, high processus spinosus muscle-intramuscular adipose tissue content, and the combination of both were significantly lower than those in the controls. These results suggest that the assessment of sarcopenia from multiple neck muscles by preoperative CT measurements may be useful in predicting the prognosis of patients with OSCC.

## 1. Introduction

The incidence of oral and pharyngeal cancer continues to increase in both men and women, with oral squamous cell carcinoma (OSCC) being the cancer with the highest mortality rate [1]. Oral squamous cell carcinoma (OSCC) is the most common type of oral cancer, accounting for 95% of all oral cancers. In the United States, from 2009 to 2018, the overall mortality rate for cancers of the oral cavity and pharynx have increased by 0.5% per year [1]. Compared to other carcinomas, oral and pharyngeal cancers are more likely to cause functional impairment, such as feeding and swallowing disorders, depending on the site and extent of surgery, and are at high risk of progressing to nutritional disorders, frailty, and sarcopenia [2,3]. Sarcopenia is defined as a condition characterized by loss of skeletal muscle mass accompanied by undesirable conditions such as functional impairment, physical disability, decreased quality of life, and death [4,5].

There have been several reports on methods to assess sarcopenia. One of the representative methods is Dual energy X-ray absorptiometry (DEXA) scanning, which has been found to accurately assess skeletal muscle mass due to its reproducibility and the advantage of being less susceptible to changes in water content [6,7,8]. However, the cost of the equipment is high, and it requires a certain amount of space for installation, which limits the number of facilities where it can be performed. In addition, the lean body mass measured by the DEXA method does not directly evaluate muscle mass and therefore cannot evaluate muscle degeneration in sarcopenia and is not suitable for evaluating changes in muscle mass in terms of sensitivity [9]. On the other hand, most studies have used computed tomography (CT) images to measure skeletal muscle mass in terms of cross-sectional area at the level of the third lumbar vertebra (L3), which correlates well with whole-body skeletal muscle mass [10]. However, abdominal CT imaging is not routinely performed in patients with squamous cell carcinoma of the head and neck [11], and imaging evaluation of the lumbar region is usually performed using ^18^F-fluorodeoxyglucose positron emission tomography/computed tomography (FDG PET/CT) taken for routine staging purposes [12].

In our previous study, we compared preoperative lumbar skeletal muscle mass at the L3 level with skeletal muscle adiposity using preoperative CT images of patients with OSCC [13]. In this study, it was reported that patients with preoperative lumbar skeletal muscle mass loss (loss of skeletal muscle quantity) and skeletal muscle adiposity (loss of skeletal muscle quality) had significantly lower disease-specific survival rates compared to controls. These results indicate that sarcopenia assessment from preoperative CT can be useful in predicting the prognosis of patients with OSCC [13].

Unfortunately, neither whole-body CT scans, including L3, nor DEXA scans are routinely performed in patients with OSCC in daily clinical practice [3], and there is a need to establish alternative methods of sarcopenia assessment to them. Therefore, it is important to validate the usefulness of sarcopenia assessment from cervical muscles, which are frequently used in routine clinical practice in patients with OSCC. In this study, we investigated whether preoperative lumbar skeletal muscle mass and adiposity in OSCC patients were associated with skeletal muscle mass and adiposity in the cervical muscles. We also investigated whether skeletal muscle mass and adiposity in the cervical muscles and their comorbidity were associated with survival rates in patients with OSCC.

## 2. Materials and Methods

### 2.1. Patients

Between January 2009 and December 2015, a total of 112 patients with primary OSCC underwent surgical treatment at Kagoshima university hospital. Of these, 10 patients did not undergo preoperative FDG PET/CT imaging and therefore excluded from the analysis. In addition, one patient was excluded because of artifacts in the patient’s CT images. Therefore, in this retrospective cohort study, 102 patients (60 males and 42 females) were enrolled and their images were evaluated.

### 2.2. Image Analysis

**Figure 1:**
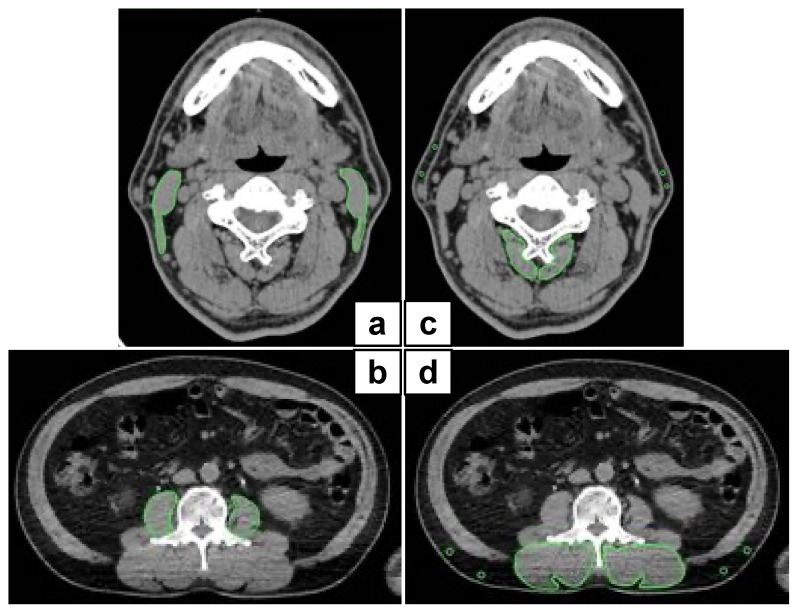
Representative cross-sectional CT images at the C3 and L3 level. (**a**,**b**) We precisely traced the subfascial outline of the multifidus and sternocleidomastoid muscles. The SCMI and PMI were determined by normalizing the cross-sectional areas to height (cm^2^/m^2^); (**c**,**d**) P-IMAC and IMAC were measured by dividing the CT values of bilateral multifidus muscles at the C3 and L3 levels by the CT attenuation values of subcutaneous fat, respectively. CT, computed tomography; SCMI, sternocleidomastoid muscle mass index; PMI, psoas muscle index; P-IMAC, processus spinosus muscle—intramuscular adipose tissue content; IMAC, intramuscular adipose tissue content.

We employed the CT component of FDG PET/CT images as a single measure of whole-body imaging to detect primary squamous cell carcinoma within two weeks before surgery. Image analysis was performed according to a previous study [14] with slight modifications. Briefly, the area of skeletal muscle was determined as areas of −29 to 150 HU. Visceral and subcutaneous adipose tissue areas were determined as areas of −150 to −50 HU. Using OsiriX v.4.0 (Pixmeo SARL, Geneva, Switzerland), we evaluated the cross-sectional areas (cm^2^) of skeletal muscle in the third cervical vertebra (C3) and the L3 region and CT values (in Hounsfield units). Sternocleidomastoid muscle mass index (SCMI) and psoas muscle mass index (PMI) were determined by normalizing the cross-sectional areas for height (cm^2^/m^2^) (Figure 1a,b). Processus spinosus muscle—intramuscular adipose tissue content (P-IMAC) and intramuscular adipose tissue content (IMAC) were determined as the region of interest (ROI) of the multifidus muscle (Hounsfield units)/ROI of subcutaneous fat (Hounsfield units). CT values of four circular ROIs on the subcutaneous fat away from the major blood vessels were measured, and the average value was employed as the ROI of the subcutaneous fat (Figure 1c,d).

### 2.3. Cutoff Values for SCMI and P-IMAC

We calculated different cutoff lines for SCMI and P-IMAC using receiver operating characteristic (ROC) curves, and selected the optimal cutoff values for each of them to classify the poor prognostic group. The cutoff values for the best estimation of SCMI and P-IMAC in males and females were determined using the Youden index. The cutoff values for SCMI in males and females were 1.831 (area under the curve (AUC) = 0.6607; sensitivity, 75.0%; specificity, 91.1%) and 1.562 (AUC = 0.7059; sensitivity, 100%; specificity, 70.6%), respectively. Based on these cutoff values, patients were classified into one of two groups: “low SCMI” and “normal SCMI”. The cutoff values for P-IMAC in males and females were −0.1375 (AUC = 0.75; sensitivity, 75.0%; specificity, 100%) and −0.1143 (AUC = 0.6568; sensitivity, 83.3%; specificity, 82.4%), respectively. Based on these cutoff values, patients were classified into one of two groups: “high P-IMAC” and “normal P-IMAC”.

### 2.4. Parameter Analysis

For all patients, the following clinicopathological variables were collected: sex, age, comorbidities, exposure to tobacco and alcohol, tumor site, TNM staging, tumor stage (according to the American Joint Committee on Cancer [AJCC] Cancer Staging Manual, Seventh Edition), treatment, follow-up duration, preoperative PMI, preoperative IMAC, preoperative SCMI, and preoperative P-IMAC. The correlations of SCMI and PMI, as well as P-IMAC and IMAC, were analyzed. Overall survival (OS), disease-free survival (DFS), and disease-specific survival (DSS) were investigated in patients classified according to preoperative SCMI and P-IMAC. To compare the survival rate with the combination of the condition of skeletal muscles, patients were assigned to 1 of 4 groups based on the cutoff values mentioned above: low SCMI/high P-IMAC, low SCMI/normal P-IMAC, normal SCMI/high P-IMAC, or normal SCMI/normal P-IMAC. A univariate analysis was performed using the log-rank test to assess the differences in survival with respect to certain covariates. Variables that significantly affected the OS, DFS, and DSS were examined by multivariate analysis according to the Cox proportional hazards regression model.

### 2.5. Statistical Analysis

The threshold for statistical significance was set at 0.05. Pearson’s correlation analyses were conducted to analyze the correlations. The Kaplan–Meier method was used to evaluate the survival curves. The survival curves were compared to the log-rank test between the two groups and the log-rank test for the trend across the four groups. Any variable identified as significant (*p* < 0.05) or that showed a value of *p* < 0.10 in the univariate analyses was considered a candidate for the multivariate Cox proportional hazards regression model. All analyses were conducted using Stata version 16 (StataCorp LLC, College Station, TX, USA) and GraphPad Prism version 6.0 for Mac OS X (GraphPad Software, San Diego, CA, USA).

## 3. Results

### 3.1. Patient Characteristics

More than half of the patients were male (58%). The mean age of the patients was 67 years old. We have included the obesity-related comorbidities; type 2 diabetes, hypertension, dyslipidemia, and cardiovascular disease which accounted for 84% of the cases. Exposure to tobacco and alcohol was identified in 48% and 38% of cases, respectively. Primary tumors involved oral mucosa sites: tongue, gingival, oral floor, buccal, palate, and lip. Advanced disease (TNM stage III and IV) at diagnosis was present in 51% of patients. Sixty patients were treated with surgery alone, and 46 patients were treated with surgery and concurrent chemoradiotherapy. The mean follow-up time was 1259 days. The mean preoperative PMI was 7.26 for men and 5.03 for women. The mean preoperative IMAC was −0.41 for men and −0.21 for women. The mean preoperative SCMI was 2.38 for men and 1.74 for women. The mean preoperative P-IMAC values were −0.32 for men and −0.23 for women. The patient demographics are summarized in Table 1.

### 3.2. The Correlations between SCMI and PMI, as well as P-IMAC and IMAC

**Figure 2:**
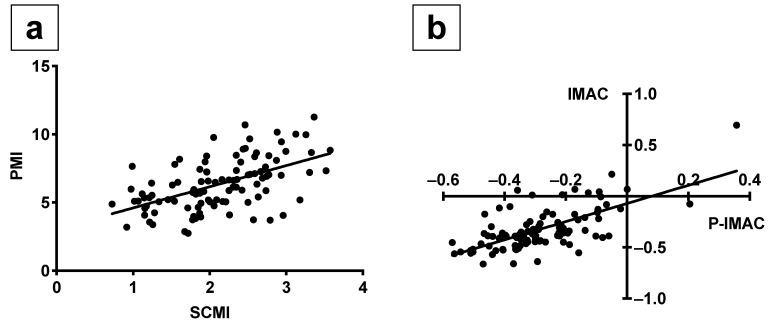
The positive correlations between SCMI and PMI, as well as P-IMAC and IMAC. (**a**) SCMI was correlated well with PMI (Pearson’s r = 0.5460; *p <* 0.0001): (**b**) P-IMAC correlated well with IMAC (Pearson’s r = 0.6689; *p <* 0.0001). SCMI, sternocleidomastoid muscle mass index; PMI, psoas muscle mass index; P-IMAC, processus spinosus muscle—intramuscular adipose tissue content; IMAC, intramuscular adipose tissue content.

There were significant positive relationships between SCMI and PMI (Pearson’s r = 0.5460; *p <* 0.0001; Figure 2a) and between P-IMAC and IMAC (Pearson’s r = 0.6689, *p <* 0.0001; Figure 2b).

### 3.3. The Survival Rates According to Preoperative SCMI and P-IMAC

**Figure 3:**
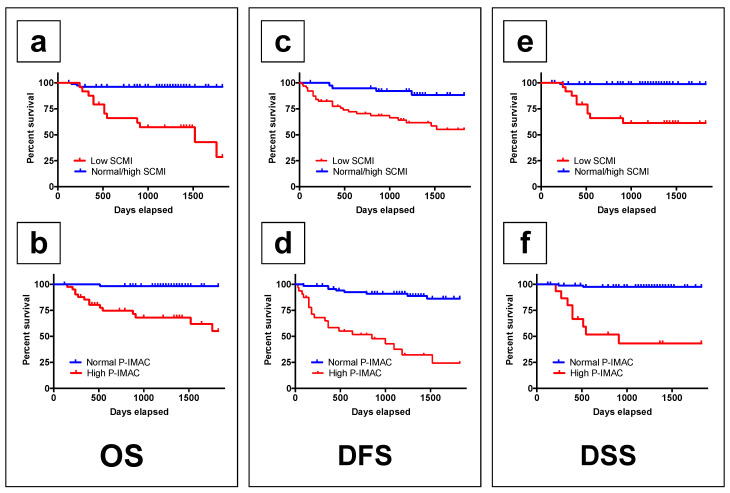
The low preoperative SCMI group and the high P-IMAC group had lower survival rates, respectively. (**a**,**b**) The overall survival rate in patients with a low SCMI and high P-IMAC was significantly lower than that in patients with a normal SCMI and normal P-IMAC, respectively (*p <* 0.0001 and *p <* 0.0001); (**c**,**d**) the disease-free survival rates in patients with a low SCMI and high P-IMAC were significantly lower than those in patients with a normal SCMI and normal P-IMAC, respectively (*p* = 0.0013 and *p <* 0.0001); (**e**,**f**) the disease-specific survival rates in patients with a low SCMI and high P-IMAC were lower than those in patients with a normal SCMI and normal P-IMAC, respectively (*p <* 0.0001 and *p <* 0.0001). SCMI, sternocleidomastoid muscle mass index; P-IMAC, processus spinosus muscle—intramuscular adipose tissue content.

The survival rates in patients with low SCMI were significantly lower than those in patients with normal/high SCMI, including OS, DFS, and DSS (*p* < 0.0001; Figure 3a–c). The survival rates in patients with high P-IMAC were significantly lower than those in patients with normal P-IMAC in terms of OS, DFS, and DSS (*p* < 0.0001; Figure 3d–f).

### 3.4. The Survival Rates According to the Combination of Preoperative SCMI and P-IMAC for Trend

**Figure 4:**
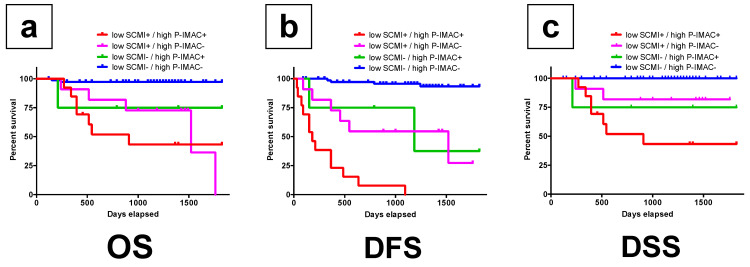
The group with lower preoperative SCMI and higher P-IMAC had a lower survival rate than the other groups. (**a**) The overall survival rate (*p <* 0.0001); (**b**) the disease-free survival rate (*p <* 0.0001); (**c**) the disease-specific survival rate in patients (*p <* 0.0001). SCMI, sternocleidomastoid muscle mass index; P-IMAC, processus spinosus muscle—intramuscular adipose tissue content.

The combination of low SCMI and high P-IMAC showed significantly lower survival rates in patients with OSCC among the groups (*p* < 0.0001; Figure 4a–c).

### 3.5. A Cox Proportional Hazards Regression Model for Survival Rates

As summarized in Table 2, we performed the multivariate analysis according to Cox regression model to confirm the prognostic factors for OS, DFS, and DSS. In the univariate analysis, the following were risk factors for OS: age (*p* = 0.016), PMI (*p* = 0.042), SCMI (*p* = 0.000), IMAC (*p* = 0.038), and P-IMAC (*p* = 0.000). The risk factors for DFS were sex (*p* = 0.012), PMI (*p* = 0.059), SCMI (*p* = 0.000), and P-IMAC (*p* = 0.000). Additionally, the following factors were associated with a statistically significant difference in DSS: age (*p* = 0.075), PMI (*p* = 0.011), SCMI (*p* = 0.001), IMAC (*p* = 0.004), and P-IMAC (*p* = 0.000). According to the multivariate Cox proportional hazards regression, only a low SCMI was statistically associated with shorter OS (HR, 13.462; 95% CI 3.136–57.791), DFS (HR, 10.179; 95% CI 3.476–29.810), and DSS (HR, 10.190; 95% CI 1.051–98.728).

## 4. Discussion

To date, few studies have examined the effectiveness of head and neck muscle measurements using CT imaging in terms of predicting total body skeletal muscle mass [15]. Because imaging at the L3 level is not routinely performed, skeletal muscle measurement at the L3 level is not always clinically applicable in OSCC patients [14]. Skeletal muscle measurement at the C3 level is a reliable and robust alternative to skeletal muscle measurement at the L3 level, allowing for a wide range of studies on the predictive and prognostic effects of sarcopenia in OSCC [16]. In the current retrospective study, we demonstrated that both the quality and quantity of muscle between the C3 and L3 levels are positively correlated with each other. These results are in line with previous reports that showed a good correlation between the C3 muscle cross-sectional area (CSA) and the L3 muscle CSA [3,17]. Additionally, we demonstrated that the survival rates in patients with low SCMI/high P-IMAC were significantly lower than those in patients with normal SCMI/P-IMAC. The combination of low SCMI and high P-IMAC demonstrated significantly lower survival rates among the groups. These results suggest that preoperative sarcopenia assessment from the evaluation of multiple cervical muscle CT scans may be useful in predicting the prognosis of patients with OSCC.

The European Working Group on Sarcopenia in Older People 2 (EWGSOP2) has recently published a representative consensus paper on the definition of sarcopenia [18]. It states that the diagnosis of sarcopenia is defined by the presence of a decrease in the quality or quantity of skeletal muscle [18]. Both the quality and quantity of the muscles are mainly used in research rather than in clinical practice for now [18]. Various methods have been used or evaluated to determine the impact of muscle quality and quantity on patients’ quality of life, but all of them need to be validated for their validity, reliability, and accuracy in the future [18]. Muscle quality is a term that refers to microscopic changes in muscle structure and composition, as well as muscle function provided per unit muscle mass [18]. Several studies have been conducted to estimate muscle quality by measuring fat infiltration into muscle, muscle attenuation, and the volume of intermuscular adipose tissue using advanced diagnostic tools such as magnetic resonance imaging (MRI) and CT [14,15]. Kitajima et al. [19] described that the multifidus muscle/fat attenuation ratio (MM/F ratio) accurately reflected the adiposization of muscle tissue with fat infiltration [19]. In the study, H&E staining revealed that the multifidus muscular tissue was infiltrated by adipose cells [19]. They hypothesized that a decrease in adipose tissue accumulation in the multifidus muscle would result in an improvement in the MM/F ratio in the multifidus muscle [19]. There are several ways to measure muscle quantity. Namely, cross-sectional area of a specific muscle group or body part, total body skeletal muscle mass, or appendicular skeletal muscle mass, although there is still debate as to which method is superior [13]. On the other hand, it has been reported that measurement of the psoas muscle at the L3 level using CT for assessing muscle quantity is useful in predicting morbidity in a variety of conditions [16].

To date, there is no universal consensus on how to assess sarcopenia in daily clinical practice [18]. Because it is still controversial as to which muscle best reflects systemic sarcopenia, and difficult to determine which one muscle is representative, the concept of using a single sentinel muscle for the diagnosis of sarcopenia was proposed by the expert groups [20,21,22]. A high IMAC indicates not only a high amount of intramuscular adipose tissue, but also a low amount of muscle mass [4]. This is because IMAC is determined as the ratio of the CT values of skeletal muscle and subcutaneous adipose tissue. On the other hand, since the psoas area estimates both muscle and intramuscular adipose tissue, PMI does not always indicate actual muscle mass because the areas of psoas muscles evaluate both muscle and intramuscular adipose tissue [23]. In our previous study, OSCC patients with preoperative low muscle quantity as defined by low PMI and low muscle quality as defined by high IMAC had significantly lower disease-specific survival than controls [13]. These results suggest that there is a substantial interaction between the preoperative comorbidity of sarcopenia and the mortality of patients with OSCC. Based on these findings, we hypothesized that the assessment of different skeletal muscle combinations could be an alternative to the assessment of muscle quality and quantity and could be the most appropriate parameter to assess preoperative sarcopenia [13]. The results of the present study partly supported this hypothesis and showed that the assessment of multiple skeletal muscle combinations in the cervical region can be used to assess preoperative sarcopenia and may also be useful in predicting prognosis.

Some limitations in the current study need to be mentioned. First, the study was retrospective in nature, with a relatively small sample size and an unequal distribution of males and females. Therefore, patient selection bias could not be completely eliminated. To confirm the findings of this study, further prospective randomized controlled studies are needed. Second, the cutoff values for SCMI and P-IMAC, as well as PMI and IMAC, are not yet well defined. However, the definition of sarcopenia in imaging assessment is still debated [24]. Furthermore, there are still no established methods or specific numerical criteria for assessing sarcopenia in imaging evaluation [24]. PMI and IMAC differ significantly between men and women, and several studies have set gender-specific cutoff values [25,26,27]. Although EWGSOP2 recommends cutoff points for simple and specific measurements [18], these cutoff values were obtained by analyzing a mainly Caucasian population, and therefore may differ from those calculated for an Asian population due to differences in body size, lifestyle, and ethnicity [28,29]. Accordingly, the validity of our proposed cutoff values needs to be confirmed in future prospective longitudinal intervention studies. Third, we did not include metabolic data in our analysis when we used FDG PET/CT images. Previous study showed that skeletal muscle FDG uptake might reflect the occurrence of inflammation as a potential pathophysiological process underlying sarcopenia [30]. On the other hand, experimental studies showed that increased skeletal muscle FDG uptake might be related to the occurrence of oxidative stress [31,32], which is known to play a role in the sarcopenic muscle. Consequently, future studies should investigate the possibility that skeletal muscle FDG uptake is related to overall survival in patients with OSCC. Lastly, assessment of the cervical muscles may not be appropriate in postoperative follow-up, especially in patients with locally advanced or recurrent OSCC. In those patients, the assessment of cervical paraspinal or sternocleidomastoid muscle may be significantly affected by neck dissection with muscle sacrifice, radical tumor resection, or radiotherapy [3,15]. Chang et al. [15] recently demonstrated a strong association between L3 muscle measurements and the masticatory-skeletal muscle index (M-SMI) assessed at the mandibular notch level. They mentioned that M-SMI assessment by head and neck CT evaluation is less susceptible to interference by lymphadenopathy and differences in measurement methods, and may be readily used as a marker of systemic SMM in patients with OSCC [15]. Further studies are needed to validate the appropriate method of evaluating sarcopenia in determining the prognosis of patients with OSCC.

## 5. Conclusions

In the current study, the quality and quantity of cervical and lumbar skeletal muscles were positively correlated preoperatively in patients with OSCC. Patients with both low quality and low quantity of cervical skeletal muscles had a significantly worse prognosis. Multivariate analysis showed that the low quantity of cervical muscle was statistically associated with shorter survival. Therefore, assessing sarcopenia by evaluating both the quality and quantity of preoperative C3 skeletal muscle mass may be useful in optimizing the treatment that contribute to improve prognosis in patients with OSCC without requiring additional imaging or patient burden. Furthermore, it may be useful in the appropriate implementation of nutritional and rehabilitative interventions aimed at improving sarcopenia.

## Figures and Tables

**Table 1:** Patient characteristics.

Characteristics	*n* = 102
Sex (male/female)	60/42
Age (years)	67 (64–69)
Comorbidities, yes	84
Tobacco/Alcohol (%)	48/38
Tumor site	
Tongue/gingival/oral floor/buccal/palate/lip	47/31/11/7/5/1
TNM staging (AJCC)	
T1/T2/T3/T4	22/54/15/11
N0/N1/N2	61/19/22
Stage	
I/II/III/IV	18/32/21/31
Treatment	
Surgery only	60
Surgery with RT/CT	42
Follow up duration	1259 (1155–1362)
Preoperative PMI	
Male	7.26 (6.81–7.70)
Females	5.03 (4.67–5.39)
Preoperative IMAC	
Male	−0.41 (−0.44–−0.38)
Females	−0.21 (−0.29–−0.14)
Preoperative SCMI	
Male	2.38 (2.22–2.53)
Females	1.74 (1.56–1.91)
Preoperative P-IMAC	
Male	−0.32 (−0.36–−0.28)
Females	−0.23 (−0.28–−0.17)

Continuous data are presented as the means (95% confidence interval (CI)).

**Table 2:** Univariate and multivariate analysis for OS, DFS, and DSS. OS, overall survival; DFS, disease-free survival; DSS, disease-specific survival; HR, hazard ratio; CI, confidence interval; PMI, psoas muscle mass index; SCMI, sternocleidomastoid muscle mass index; IMAC, intramuscular adipose tissue content; P-IMAC, processus spinosus muscle—intramuscular adipose tissue content.

Variable	OS				DFS				DSS			
	Univariate	Multivariate			Univariate	Multivariate			Univariate	Multivariate		
	*p*	HR	95%CI	*p*	*p*	HR	95%CI	*p*	*p*	HR	95%CI	*p*
Age	0.016	1.035	0.981–1.092	0.206	0.282				0.075	0.994	0.930–1.062	0.864
Sex	0.934				0.012	1.079	0.394–2.950	0.881	0.254			
Comorbidities	0.792				0.195				0.546			
PMI												
Low	0.042	1.150	0.378–3.497	0.805	0.059	0.925	0.386–2.211	0.861	0.011	2.986	0.605–14.718	0.179
SCMI												
Low	0.000	13.462	3.136–57.791	0.000	0.000	10.179	3.476–29.810	0.000	0.001	10.190	1.051–98.728	0.045
IMAC												
High	0.038	2.092	0.656–6.663	0.212	0.172				0.004	2.464	0.672–9.030	0.045
P-IMAC												
High	0.000	1.081	0.311–3.752	0.902	0.000	5.857	2.088–16.425	0.001	0.000	4.815	0.761–30.442	0.095

## Data Availability

Data available on request due to restrictions of ethical policy.

## Abbreviations

AJCC	American Joint Committee on Cancer
AUC	Area under the curve
C3	Third cervical vertebra
CI	Confidence interval
CSA	Cross-sectional area
CT	Computed tomography
DEXA	Dual energy X-ray absorptiometry
DFS	Disease-free survival
DSS	Disease-specific survival
EWGSOP2	European Working Group on Sarcopenia in Older People 2
FDG PET/CT	^18^F-fluorodeoxyglucose positron emission tomography/computed tomography
HR	Hazard ratio
IMAC	Intramuscular adipose tissue content
L3	Third lumbar vertebra
M-SMI	Masticatory-skeletal muscle index
MM/F	Multifidus muscle/fat
MRI	Magnetic resonance imaging
OS	Overall survival
OSCC	Oral squamous cell carcinoma
P-IMAC	Processus spinosus muscle—intramuscular adipose tissue content
PMI	Psoas muscle mass index
ROC	Receiver operating characteristic
ROI	Region of interest
SCMI	Sternocleidomastoid muscle mass index
